# Education outside the classroom in Germany: An interdisciplinary multi-methods Study Protocol

**DOI:** 10.1371/journal.pone.0337524

**Published:** 2026-06-23

**Authors:** Anna Brandmeier, Melissa Börner, Theres Mühlberg, Leonie Schulz, Lukas Kleinhenz, Theresia Endriß, Stephan Ellinger, Jan Schmid-Ellinger

**Affiliations:** 1 Associate Professorship of Didactics in Sport and Health, School of Medicine and Health, Technical University of Munich, Munich, Germany; 2 Urban Productive Ecosystems, School of Life Sciences, Technical University of Munich, Munich, Germany; 3 Chair of Special Education I, Education for People with Learning Disabilities, Faculty of Human Sciences, Julius-Maximilians-University Wuerzburg, Wuerzburg, Germany; PNG National Research Institute, PAPUA NEW GUINEA

## Abstract

**Background:**

Schools are a central place in the lives of both children and teachers, with the potential to counteract current challenges such as a physical and emotional disconnection from nature, insufficient physical activity, and increased social isolation. Concepts like Education Outside the Classroom (EOtC) offer a variety of approaches and are already established in the curriculum in other countries, while in Germany EOtC is still in the early stages of implementation. Previous studies have focused on the effects of EOtC on students, with little attention paid to teachers, environmental perception, or behavioural analyses. This study aims to evaluate the impact of EOtC on teachers’ stress level, teachers’ well-being and physical activity, pupils’ cognitive performance, school-related well-being, basic psychological needs and classroom behaviour. In addition, this study investigates how the biodiversity of selected outside learning environments affects pupils’ and teachers’ nature connectedness, appreciation of nature and species knowledge.

**Methods:**

This study protocol describes an exploratory, quasi-experimental, interdisciplinary study conducted in a total of 33 schools throughout Germany. Data collection will be carried out from September 2025 to November 2026. The first results of the data collection are expected in October 2026. It includes approximately 50 EOtC teachers, 50 non-EOtC teachers, 50 EOtC classes, and 18 non-EOtC classes with for intra- and interindividual comparisons. Data collection covers aspects from the fields of health, ecology, school pedagogy, and sociology and includes both pupils and teachers as target groups, as well as the various outdoor learning environments. For pupils and teachers, the study examines the effects of EOtC on nature connectedness, appreciation of nature, and plant and animal species knowledge. This is assessed twice per school year with questionnaires and compared to control classes within the same schools. In addition, for pupils, cognitive performance, measured by electroencephalography (EEG), school-related well-being, and basic psychological needs – both assessed via questionnaires – will be analysed. For teachers, stress levels, emotional well-being, and physical activity will be assessed using salivary cortisol samples, accelerometery, and questionnaires on both EOtC and regular teaching days, enabling intraindividual analyses. Moreover, teaching characteristics will be documented, and video-based analyses of social interactions between teachers, pupils, the outdoor learning environment, and teaching objects are conducted. Finally, biodiversity at the outdoor learning sites and their effects on the other study variables (e.g., nature connectedness, appreciation of nature, species knowledge and well-being of pupils and teachers) will be statistically analysed.

**Discussion:**

This interdisciplinary study design provides a realistic analysis of the potential effects of EOtC on pupils, as well as teachers, while minimizing interference with the regular teaching process. A strength of this study is the consisted inclusion of teachers as a target group, as well as the ecological perspective and the use of innovative survey methods such as the recording of biodiversity, the video analysis of interactions and the analysis of lesson characteristics in the context of EOtC. This study will thus contribute to closing central research gaps in the German context and provide valuable insights for international EOtC research.

## 1. Introduction

Children and adolescents in Germany, as in other Western countries, face a variety of social and health-related challenges, such as insufficient physical activity (PA) [1], declining mental health [2] and increasing social isolation [3]. Additionally, an increasing alienation from natural spaces has been observed [4,5], which is causing a decrease in the potential positive effects of nature experiences and opportunities for social interaction and PA. Schools represent a central living environment for children and adolescents: pupils spend an average of 20–30 hours per week in class [6], plus additional time spent on homework or extracurricular activities. Thus, schools could be an effective starting point to counteract the loss of nature experiences – often referred to as the ‘extinction of experience’ – as well as related health and social challenges. At the same time, the teachers also often work well beyond the mentioned hours of classroom teaching [7,8]. Teachers are increasingly exposed to high workloads, time pressure, and long periods of sedentary behaviour (SB), while at the same time having to deal with the social and emotional dynamics of their pupils, as well as their own [8,9]. In response to these growing demands for both pupils and teachers, there is increasing interest in educational approaches that address and improve the deficits regarding nature connectedness, PA, social interaction and psychosocial well-being in both pupils and teachers. Although there are many different concepts, add-on programs often fail due to limited sustainability. Instead, concepts that can be seamlessly integrated into everyday school life, are preferred. One of these concepts with diverse social, pedagogical, health, and nature-related potential is *Education Outside the Classroom* (EOtC). This concept provides a shift in the learning environment, which can provide valuable new impulses for both pupils and teachers. Such temporary spatial changes have been shown to foster well-being, support social interaction and, depending on the setting, strengthen awareness and appreciation for the environment [10,11].

A wide range of concepts and practices, such as *outdoor education, forest school, friluftsliv* and *learning outside the classroom* have emerged internationally and tap into the potential offered by temporary changes of location. Although these concepts share the idea of relocating educational activities outside school buildings, they differ in many ways, including pedagogical emphasis, cultural background and contextual adaptation [12,13], as well as their structure, goals and implementation style. Within this diverse field*,* EOtC has developed into a curriculum-aligned teaching context that takes place outside the traditional classroom in natural, cultural, historical, or urban environments and is embedded in the regular school schedule [11,14]. In this context, EOtC should not be confused with occasional school trips or project days, but rather understood as a structured, ongoing part of weekly or biweekly lessons lasting at least four hours, which are usually conducted over several consecutive months or longer, with the regularity of this format expected to reinforce potential effects. While EOtC is already an integral part of the teaching culture in some countries, such as Denmark, Norway, Sweden and New Zealand, there are only a few schools in Germany so far that implement the concept. Because of its characteristics of regularity and long-term nature, EOtC has the potential to cause sustainable changes. Previous research showed that EOtC can have positive effects on pupils’ social well-being [11,15], stress regulation [16,17], intrinsic learning motivation [18,19], understanding of nature and environmental attitudes [20,21], PA [22,23] and academic performance, such as reading or writing skills [24]. These results suggest that EOtC may have the potential to combine health promotion, learning support, social interactions, and positive experiences in nature.

Even though existing research already highlights important effects of EOtC on pupils, further questions remain unanswered. For example, how different EOtC environments may influence pupils’ knowledge, basic psychological needs, nature connectedness and social interactions, as well as those with nature. It is important to understand how pupils interact not only with the environment but also with their classmates, as these interactions influence not only individual outcomes but also the overall learning environment. Furthermore, most research to date has focused primarily on the impact of EOtC on pupils, while teachers and the demands of their work environment have received comparatively little attention.

Therefore, this EOtC study is an interdisciplinary research project, aiming to investigate the potential effects of regular EOtC on health-related, social, pedagogical, and ecological outcomes in German schools and to examine how these outcomes are interrelated across these domains where possible. Given this thematic range and methodological approach, the study addresses a set of research questions structured across four key domains, which are outlined below.

Health-related research questions:

1. Can intra-individual differences in diurnal cortisol dynamics, specifically the subsequent decline throughout the school day, be observed in teachers on EOtC days compared to regular indoor teaching days?
2. What differences in teachers’ hedonic well-being, particularly in terms of positive and negative affects, can be observed on EOtC days compared to regular indoor teaching days?
3. What differences in teachers’ physical activity and sedentary behaviour can be observed on EOtC days compared to regular indoor teaching days?
4. To what extent is higher work-related quality of life (WRQoL) observed among teachers participating in EOtC compared to those teaching exclusively in indoor settings?
5. How do children’s EEG patterns related to attention, stress, and emotional regulation differ between EOtC and regular indoor teaching days?
6. Is there another dimension in the construct of BPNs that addresses the fulfillment of the need relatedness through a non-social factor, namely nature relatedness?
7. Which effects do BPNs and/or nature relatedness, if considered a separate construct, have on children’s school-related well-being?

Pedagogical research questions:

1. Is there a difference in the extent of the applied teaching principles between teaching in EOtC and classroom teaching?
2. To what extent are the different teaching principles applied during teaching in EOtC?
3. What kind of changes in teaching, learning and “doing school” occur when teachers and students leave the familiar classroom environment to conduct lessons outdoors in nature?
4. How do teachers respond in their instructional and pedagogical practice to the challenges of conducting school lessons outside the classroom?
5. Are there differences in student behavior between teaching in EOtC and classroom teaching, specifically in the way they participate in lessons or disengage from educational interactions? What role does nature play in this context?
6. How do children with learning disabilities and behavioral emotional disorders respond to EOtC?
7. Does EOtC offer the potential to rediscover school as an appealing learning space, which ultimately may restore and promote commitment to school-based learning?

Ecological research questions:

1. Does EOtc lead to higher species knowledge in pupils and teachers?
2. Does EOtC lead to higher connectedness to nature in pupils and teachers?
3. Does EOtC lead to higher appreciation of nature in pupils and teachers?
4. Does higher biodiversity of outdoor learning sites lead to higher species knowledge, connectedness to nature and appreciation of nature in pupils and teachers?
5. Does higher structural complexity (e.g., higher forest development stages and higher deadwood amount) of outdoor learning sites lead to higher species knowledge, connectedness to nature and appreciation of nature in pupils and teachers?

With this interdisciplinary and multi-methods approach, the project represents a novelty in EOtC research in Germany and enriches international research on EOtC by addressing vital questions. Given the complexity of EOtC as an educational and environmental intervention, an interdisciplinary approach like this is essential and calls for a transparent description of the methodological approach. This study protocol describes the design, methods, and analysis strategies for this comprehensive, interdisciplinary project.

## 2. Design & Methods

### 2.1. Study aims and study design

This interdisciplinary, Germany-wide study examines a) the multifaceted impacts of EOtC by combining expertise from the fields of public health, ecology, pedagogy, special needs education and social sciences, and b) possible connections between constructs across these domains in the context of EOtC. The study follows a parallel multi-strand research approach, combining independent quantitative and qualitative sub-studies within an overarching framework. This exploratory, quasi-experimental study will be conducted in schools throughout Germany.

#### 2.1.1. Aims and design of the health-related study component.

The health-related objective of this study is to examine how regular participation in EOtC is associated with psychological, physiological, and behavioural indicators of health among teachers and pupils. Regarding teachers, this study component investigates whether EOtC contributes to improved reduction of physiological stress, emotional well-being, and higher PA by comparing EOtC and classroom teaching days within individuals. Additionally, WRQoL is assessed between teachers who regularly implement EOtC and those who exclusively teach inside the classroom. Regarding pupils, the study explores potential links between EOtC and cognitive functioning, emotional regulation, and stress. It also investigates basic psychological needs (BPN) and nature connectedness as distinct psychological correlations of school-related well-being.

The health-related measurements of the project follow a cross-sectional, quasi-experimental design and are embedded in a broader, cross-disciplinary mixed-methods evaluation, which will be further explained later.

#### 2.2.2. Aims and design of the ecology-related study component.

To assess the role of EOtC in *Education for Sustainable Development* (ESD) the key components knowledge, nature connectedness and appreciation of nature will be assessed, as they are known to promote pro-environmental behaviour and attitude [25,26]. The aim of the ecological component of this study is therefore to understand how regular EOtC improves the nature connectedness and appreciation of nature in pupils and teachers, as well as the knowledge of species. In addition, biodiversity and structural characteristics of the learning sites will be systematically recorded, providing the basis for exploring how variation in ecological richness related to educational and health-related outcomes.

The ecological questionnaires are collected in longitudinal surveys twice per school year and are also included in the cross-disciplinary, mixed-methods evaluation. In addition, the biodiversity of the outdoor learning areas at selected schools will be mapped once during the school year and combined with data from ecological, health and sociological surveys.

#### 2.2.3. Aims and design of the social interaction and special needs-related study component.

The pedagogical component of this study focuses on the types of interactions between teachers, pupils, and learning objects during EOtC lessons, with particular attention to the promotion of pupils with special educational needs. Using a reconstructive, qualitative analysis of video recordings from both classroom teaching and EOtC, this study investigates how teaching and learning processes emerge, or fail to emerge, depending on the environment.

On the teacher’s side, particular attention is paid to how they engage with the changed spatial conditions of EOtC. The core research question is whether changes occur in teachers’ instructional behaviour when moving from a structured classroom environment to an unstructured outdoor learning environment and how these changes look like. Furthermore, the study aims to identify different teaching styles and strategies that may either promote or hinder students’ learning processes in EOtC. On the pupils’ side, the focus lies on the nature of their interactions with teachers, peers, and learning materials. The guiding question here is whether leaving the structured classroom environment results in qualitative changes in these interactions. As an additional perspective, resonance theory [27] will be used to explore whether EOtC fosters moments of resonance and reduces experiences of alienation, particularly among pupils who often struggle to engage in classroom lessons.

In this context, resonance is defined as a reciprocal relationship between subject and world, comprising four key dimensions: (a) being affected, where the subject feels addressed by the world and is moved by something; (b) an agentic response, in which the subject responds with their own voice and a sense of agency; (c) transformation, referring to a process of mutual transformation (“Anverwandlung”, German) between subject and world that results in change; and (d) unavailability (“Unverfügbarkeit”, German), meaning that this process is neither predictable nor fully controllable by either side [27].

This model (particularly dimensions a–c) can be applied to school-based learning and ideal-typical learning phases to describe barriers within the learning process [28]. The dimensions of resonance will be used as an analytical framework for empirical video data, functioning as sensitizing concepts [29], in order to reconstruct interaction patterns and teaching–learning processes from a resonance-theoretical perspective.

Furthermore, this study aims to identify core pedagogical principles and structural features which characterize EOtC in contrast to traditional classroom instruction.

### 2.2. Setting – the German school system

Schooling in Germany begins with primary education (grades 1–4), typically starting at the age of six. This is followed by the secondary system, where pupils are either allocated to different school types based on their academic performance or parents are free to choose a school for their child, depending on the regulations of the respective federal state. Although education policy and curricula are the responsibility of the individual federal states, overarching educational standards are set by the Standing Conference of the Ministers of Education and Cultural Affairs (Kultusministerkonferenz) to ensure national comparability. Schools can be publicly, privately, or denominationally funded, but are all legally obliged to implement the state-specific curricula and educational guidelines [30]. Therefore, both primary and secondary schools, as well as special needs schools throughout Germany can be included in the sample, provided they meet the inclusion criteria.

### 2.3. Recruitment process and participants

**Fig 1:**
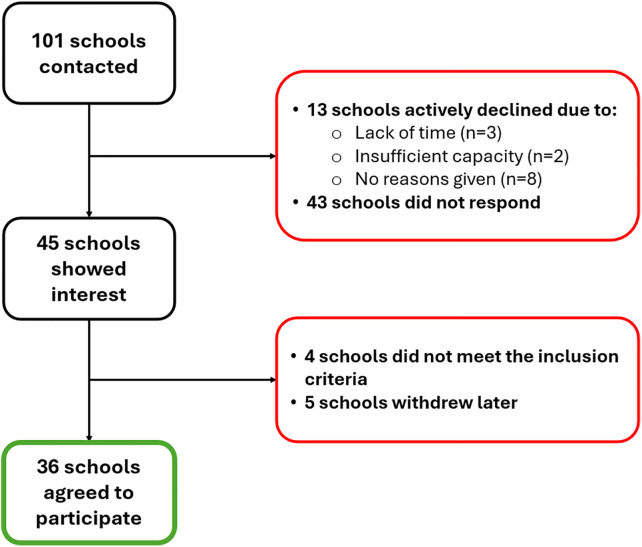
Stages of recruitment.

As mentioned above, EOtC is defined as regular, curriculum-based teaching, which is conducted in a natural, cultural, historical or urban environment [11,14]. This definition builds the base for inclusion and exclusion criteria when identifying schools, classes and teachers to participate in this study. Schools were included in the study if EOtC was carried out for at least four hours every two weeks. This threshold was chosen to ensure that participating classes engage in EOtC for approximately one full day per week in the case of elementary school and at least half a school day in the case of secondary schools. No additional exclusion criteria were defined. The frequency is intended to emphasize that EOtC is not a sporadic activity, but rather a consistent and integral element of the pupils’ and teachers’ routine. Schools in all of Germany’s federal states were contacted in order to represent as many federal states, school types and associated learning locations as possible. A total of three different sources of contact were used to recruit schools for data collection: Some contacts already existed to individual schools implementing EOtC in Germany through previous projects and conference attendance by individual scientists. Similar collaborations with schools already existed with the external project partner, Schutzgemeinschaft Deutscher Wald (SDW). In addition, an internet search was carried out for schools which have implemented the concept of EOtC. In the recruitment phase from beginning of January to the end of July 2025, 101 schools were initially contacted. Of these, 45 schools showed interest. 13 schools actively declined, citing reasons such as lack of time, insufficient capacity or no specific reason. Four schools did not meet the inclusion criteria. 43 schools did not respond at all. Five schools withdrew after initially agreeing to participate, resulting in a final sample of 36 schools. A flowchart mapping the various stages of recruitment, number of excluded and included schools, as well as reasons for inclusion/exclusion and the numbers of participating pupils and teachers is shown in Fig 1. To obtain a precise overview of the classes, pupils and teachers involved, network meetings were held at which the planned surveys were first presented, and the interest of the individual teachers was then queried. Emails were sent to the schools that were unable to attend the meetings to obtain a final sample size. In addition to EOtC classes and teachers, corresponding control classes and teachers were also sought at each participating school to include them in the data collection as both intra- and inter-individual comparisons are planned in the study design.

**Fig 2:**
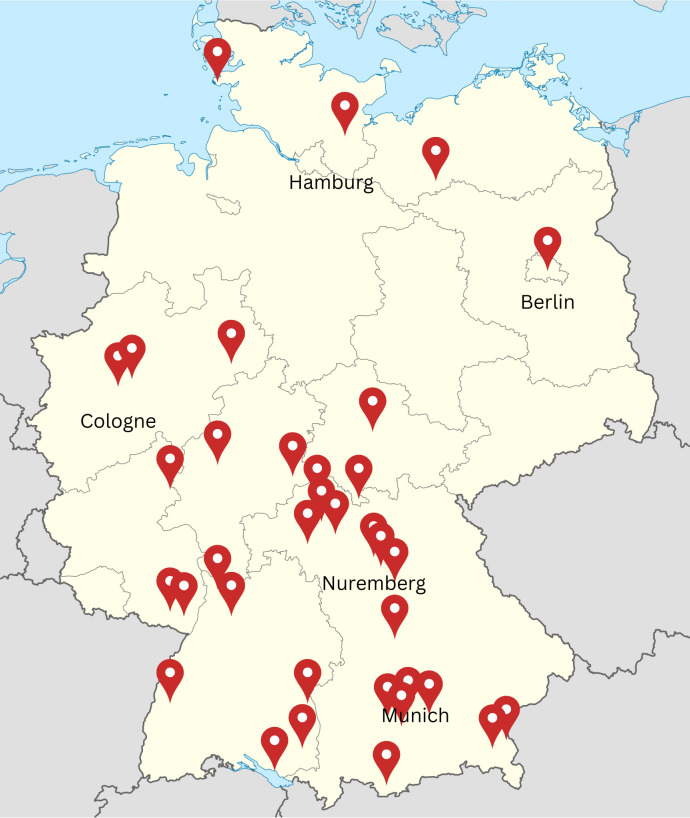
Schematic representation of the study locations in Germany. Base map adapted from Wikimedia Commons (public domain).

This results in expectedly 36 schools with approximately 50 EOtC teachers, 50 non-EOtC teachers, 50 EOtC classes, and 18 non-EOtC classes (Fig 2). All participants provide written informed consent prior to taking part in the study. Each participant receives detailed written information about the study’s aims, procedures, and data protection measures. In the case of child participants, written consent is obtained both from the children themselves (age-appropriate assent) and from their parents or legal guardians. All data are pseudonymized using individual participant codes, which are self-generated by the participants to ensure confidentiality. The research team has no direct access to personal identifiers.

### 2.4. Data collection and measurements

All data will be collected during the school year, specifically from September 2025 to November 2026. The first results of the data collection are expected in October 2026. Both cross-sectional and longitudinal data will be collected during this time. While some data collections are carried out in isolation due to their content focus, surveys are bundled wherever possible to reduce redundancy and the effort required of respondents. During school recruitment, information was collected about whether EOtC was implemented all year-round. This revealed that some schools reduce or suspend EOtC during late autumn and winter. To account for these variations and capture seasonal effects, data collection is scheduled for early autumn, spring and summer. Background information from both teachers and parents of participating pupils is gathered at the start of the school year through an online survey.

Health-related data collection is structured by target group. To minimize the response burden, data will be collected on different occasions during the school year. Among teachers, the WRQoL is administered during the first half of the school year. In the second half, physiological stress and PA as well as SB are assessed on several consecutive days, in addition to the linked questionnaires on subjective well-being and sleep. For pupils, the data collection of questionnaires, electroencephalographic (EEG) measurements and teaching observations is spread over the entire school year, with seasonally sensitive instruments scheduled accordingly.

The ecological surveys are carried out at the beginning and end of the school year. The selected questionnaires are collected in a combined form from both pupils and teachers. Attention is paid to the pupils’ knowledge of species being asked at the beginning to avoid a loss of concentration due to previous questionnaire parts, since knowledge-based queries call for a “correct” answer.

The recordings for the video and audio analysis of the social interaction component are spread throughout the school year and involve both pupils and teachers at three selected schools, all of which are experiencing EOtC for the first time. Classroom observations also take place at these selected schools throughout the school year.

### 2.5. Measures Health

#### 2.5.1. Teachers’ background and self-efficacy.

To collect the necessary background data on teachers, both their teaching experience in years and their self-efficacy will be assessed. Work experience is recorded by asking a single question about the number of years of teaching experience in this position. The *Scale for Teacher Self-Efficacy* (STSE) [31] is used to measure the self-efficacy in their profession. It’s the validated German questionnaire of the short version of the ‘Teachers’ Sense of Efficacy Scale’ by Tschannen-Moran and Hoy [32]. The questionnaire consists of three scales, inquiring about instructional strategies, class management and student motivation. Each scale comprises four items and will be rated on a nine-point Likert-scale, ranging from not at all convinced to completely convinced.

#### 2.5.2. Teachers’ physiological stress.

Physiological stress will be measured by salivary cortisol, a validated biomarker of the hypothalamic–pituitary–adrenal axis (HPA) activity that has previously been applied in educational settings [17,33,34]. Saliva samples will be collected by the teachers themselves using Salivettes^®^ (Sarstedt, Germany), with individual sampling kits provided to all participants to ensure standardized collection. To account for intra-individual variability in cortisol secretion, a repeated-measures design will be used. The sampling will be conducted on four separate days, comprising two days of EOtC sessions and two days of classroom teaching. On each day, the cortisol awakening response (CAR) will be measured as an individual baseline reference. This includes one saliva sample immediately upon awakening and a second sample 30 minutes later for men and 45 minutes later for women to capture the peak cortisol level. The adjusted timing accounts for gender-specific differences in the temporal dynamics of the cortisol response to awakening [35,36].

In addition, three daytime samples will be collected on each measurement day at standardized time points (8:00 AM, 10:15 AM, and 12:30 PM) to determine the diurnal cortisol pattern and allow comparisons across days and conditions. After collection, samples will be stored during the day in the provided insulated bag and frozen in a household freezer in the evening prior to collection by study staff. To account for potential confounding effects of sleep duration and sleep quality on cortisol secretion, sleep-related variables will be assessed separately.

#### 2.5.3. Teachers’ sleep behaviour.

To account for potential effects of sleep on cortisol secretion and diurnal patterns, three core aspects of sleep will be assessed: subjective sleep quality, sleep duration, and individual chronotype. Sleep quality will be assessed using the validated *Single-Item Sleep Quality Scale* (SQS), which is a reliable tool for capturing self-reported sleep quality in a single question, using a scale from 0 (terrible) to 10 (excellent) [37]. Sleep duration will be assessed by asking participants to report the total number of hours they slept during the previous night, as well as their approximate time of falling asleep (defined as the time when lights were turned off and no further activities such as using electronic devices were done) and their time of waking up. Individual chronotype will be measured using the *reduced Morningness–Eveningness Questionnaire* (rMEQ), a validated instrument that has been translated into German and enables a reliable classification of the chronotype [38]. It comprises five questions with response options covering different periods in the morning and evening.

#### 2.5.4. Teachers’ emotional well-being.

Teachers’ emotional well-being and affective state are assessed using the German version of the *Positive and Negative Affect Schedule* (PANAS) [39,40]. The PANAS is a widely used, validated self-report instrument that distinguishes between positive and negative affect as two independent dimensions of emotional experience. Its psychometric properties have been confirmed in various populations, including occupational and educational settings [41,42]. The questionnaire consists of 20 items – ten for positive affect and ten for negative affect – rated on a five-point Likert scale from “not at all” to “extremely.” The PANAS has been used in studies investigating teachers’ emotional states in everyday school life, particularly in relation to occupational stress and well-being [43,44], which allows for comparisons.

#### 2.5.5. Teachers’ physical activity and sedentary behaviour.

Teachers’ PA and SB will be objectively assessed using ActiGraph wGT3X-BT accelerometers in accordance with current best practice [45]. The devices are worn on an elastic belt positioned at the right hip at the level of the anterior superior iliac spine and continuously record movement and sedentary time. Participants will wear accelerometers on both EOtC days and classroom teaching days to enable comparisons between settings. The devices provide information on the intensity and duration of PA as well as SB during the day. Activity intensity will be classified according to established cut-points for healthy adults [46,47]. Teachers will wear the accelerometers over a period of four days, including two days with EOtC, in order to obtain a comprehensive activity profile during the school week, as was done in previous EOtC research projects regarding children’s PA [48]. In addition, these measures will be explored in relation to cortisol patterns, given established associations between PA, SB, and HPA axis regulation.

#### 2.5.6 Teachers’ work-related quality of life (WRQoL).

Teachers’ WRQoL will be assessed using the *Copenhagen Psychosocial Questionnaire III* (COPSOQ III). The internationally established instrument has been translated into German and validated for use in occupational health research [49]. The COPSOQ III is widely applied in studies on work-related psychosocial factors and health outcomes, enabling comparability of findings within the educational sector [50,51]. A profession-specific version of the questionnaire will be used, including a general section on workplace characteristics and a teaching-specific section covering work tasks, emotional demands, subjective evaluations of the occupational situation, and health-related aspects of the work environment [51].

#### 2.5.7. Pupils’ socioeconomic status and background.

To assess pupils’ socioeconomic background, parents will be asked to report their socioeconomic status (SES) based on the three dimensions of education, occupation and income. An index is created by adding up the points assigned based on the parents’ statements regarding the dimensions mentioned. Based on the index, a categorization of the children into different status groups will be undertaken. Both the questionnaire and the analysis will be based on the procedure of the KiGGS study [52], creating comparability to a nation-wide study on health of children and adolescents. Additionally, children aged 11 and older will be asked to report their subjective social status (SSS) using the German version of the MacArthur Scale for children [52]. This validated instrument presents a visual ladder to help individuals indicate their perceived position within the social hierarchy, with higher placements reflecting a higher perceived social standing [53]. The measure is recently gaining more attention due to its possible own relevance to the individual health status, especially concerning psychological health outcomes such as psychological well-being [54,55].

In addition, pupils will be asked to report their age and gender, as well as provide information on environmental and lifestyle factors that may indicate greater exposure to nature in everyday life. These include whether they have access to a balcony or garden at home, or whether they own a pet. Pupils will also be asked how long they have participated in EOtC and whether they regularly spend time outdoors after school, as time spent in nature may influence outcomes such as nature connectedness and well-being [56,57].

#### 2.5.8. Pupils’ cognitive performance and stress level during class.

To assess pupils’ cognitive performance, electroencephalography (EEG) is used to provide objective, real-time insights into neural processes associated with attention, cognitive load, and engagement.

For this purpose, a mobile EEG system (EPOC X, EMOTIV) is used, which has been successfully applied in previous research investigating cognitive processes in school contexts [58–60]. Growing evidence supports the use of the EMOTIV EPOC in capturing neural responses to cognitive demands in children. It has been employed to detect intervention effects on brain activity during attention tasks [58], to assess learning and emotional states via spectral power analysis [59], and to map brain connectivity during tasks requiring attention and creativity [60]. These findings support the validity of using the EPOC X system for EEG measurements related to attention and cognitive load in children. The EEG device features 14 channels for capturing neural activity, enabling a differentiated analysis of pupils’ cognitive states. To control for individual variability, data are collected intra-individually, with each pupil serving as their own control. EEG recordings are conducted under comparable contextual conditions, namely the same subject, the same teacher, and the same time frame, both during EOtC sessions and classroom teaching.

To control for potential stress induced by wearing the EEG device itself, children’s perceived stress is assessed using a visual analogue scale (0 = “not stressed at all” to 10 = “extremely stressed”). Similar approaches have been applied in studies investigating the stress-reducing effects of multisensory environmental stimuli [61,62].

#### 2.5.9. Pupils‘school-related well-being.

School-related well-being will be assessed using the German version of the *EPOCH-G-S* questionnaire [63], a self-report instrument developed to capture multidimensional aspects of adolescent well-being in the school context. It consists of 19 items across five subscales – Engagement, Perseverance, Optimism, Connectedness, and Happiness – each rated on a five-point Likert scale from “not true at all” to “completely true.” The EPOCH-G-S has shown initial validity in educational research, particularly in studies exploring associations with academic performance, achievement motivation, and self-esteem [64,65]. Its multidimensional and context-specific structure allows for a differentiated assessment of well-being that addresses school-related experiences and psychosocial outcomes. Since the validated age range for the scale is 10–18 years and the target sample in this study comprises children aged 8 years, a cognitive pretest with structured interviews is conducted to assess the comprehensibility of the items and to ensure content validity for younger age groups.

#### 2.5.10. Pupils’ fulfillment of Basic Psychological Needs and views on nature relatedness as a new Basic Psychological Need.

The satisfaction of pupils’ BPN within the educational setting will be assessed using the *Basic Psychological Needs in the Classroom Scale* (BPN-CS; [66]. The 17-item scale includes four subscales – autonomy, competence, relatedness, and novelty satisfaction – rated on a five-point Likert scale from “strongly disagree” to “strongly agree.” It has been validated for use in children aged 8–13 years. The questionnaire offers a structured, age-appropriate measure of need satisfaction within the classroom context and has been used in comparable studies [67–69].

To examine whether the concept of nature relatedness may represent an additional individually perceived psychological need in the context of Basic Psychological Needs Theory [70–72], focus group interviews will be conducted. These interviews will follow a semi-structured guide and aim to generate insights that will inform potential development and inclusion of a new subscale in the translated version of the BPN-CS.

### 2.6. Measures Ecology

#### 2.6.1. Structure and features of outside learning environments.

At each chosen EOtC site, biodiversity is recorded in the form of avian species richness, tree and shrub species richness and characteristic spring ephemerals species richness. To describe structural complexity, forest development stages according to Hilmers et al. (2018) are documented where applicable (in forests) and the amount of deadwood is quantified [73]. Each site is additionally evaluated using a standardized ecological value score, adapted from the Bavarian Compensation Ordinance (BayKompV) [74]. Where applicable, canopy cover is estimated based on upward-facing photographs, from which the percentage of tree crown coverage is calculated using image analysis. Furthermore, the type and number of human-made habitats resulting from EOtC activities (e.g., insect hotels, bird nest boxes, hedgehog piles and more) are recorded at each site. This multi-faceted ecological assessment provides the basis for categorizing the learning sites according to their structural and species richness, serving as a foundation for subsequent analysis of their potential influence on pupils ‘and teachers‘ nature connectedness, species knowledge, well-being and experienced moments of resonance.

#### 2.6.2. Pupils’ and teachers’ nature connectedness.

Both pupils’ and teachers’ nature connectedness will be measured using the *Illustrated Inclusion of Nature in Self Scale* (IINS) [75]. The IINS is a single-item, pictorial measure validated and is used for children aged 9–14, including those with learning difficulties [76,77]. Due to its single item structure and illustrated form, it is easy to understand and is suited for use in different cultures and age groups as well as for people with a lower level of imagination or abstraction. However, because of its simplicity, it cannot be tested for its internal consistency [75,78]. Therefore, the *Nature Connection Index* (NCI) is also included in the questionnaire. The NCI is a standardized tool, which is available in German language. It includes six items and shows long-term changes with stable results in both children and adults [79,80]. In this questionnaire, participants assess six statements using a seven-part Likert scale to specify their level of agreement or disagreement. For making sure that children understand the levels of agreement, coloured smileys are added to the scale.

#### 2.6.3. Pupils’ and teachers’ appreciation of nature.

The shortened, German version of the *2-MEV* (2-major environmental values model) questionnaire, which was validated by Randler and colleagues in 2024, was included in our survey [81]. Similar to the NCI, agreement levels are stated about six different statements by participants on a five level Likert scale, including smileys for younger participants.

#### 2.6.4. Pupils’ and teachers’ species knowledge.

Since it has been shown that knowledge of local species increases children’s appreciation of these species [82] a combination of native species found throughout Germany was selected for this study, excluding mammals. Identification rates of mammals have been shown to be highest when species knowledge was tested in the past, being the most phylogenetic similar to humans and present in children’s books and television [83]. Therefore, the query focuses on other species, covering trees, shrubs, flowering plants, insects, one of each a nematode and a spider as well as two bird species, showing coloured pictures of the respective species in an open-ended item format. For each species, one photograph as well as one or two sketches are shown to ensure a detailed depicture of the species. Participants can gain 1 point for each species named, up to a maximum of 25 points, and 0.5 points if they cannot give the exact name of the species but can only indicate a higher taxonomic group.

### 2.7. Measures Social Interactions and Special Needs

#### 2.7.1. Reconstructive teaching and learning analysis.

In contrast to research based on subsumptive logic, reconstructive classroom research seeks the structure of meaning inherent in protocols of events. It is based on written records of teaching practice and aims at uncovering latent structures of meaning [84] through interactional analysis [85], documentational analysis [86], in-depth hermeneutic analysis [87], and objective hermeneutic analysis [88,89]. For this reason, weekly EOtC lessons at individual schools are recorded using three cameras installed at the learning location. In addition, at least two lessons per week are videotaped in the classroom, whereby care is taken to ensure that the lessons are taught by the same teacher. This allows differences in teaching and learning processes between EOtC and classroom teaching to be systematically reconstructed. Special attention is given to pupils with special educational needs, as most of the participating classes for video recording are from special-needs schools, making it possible to examine how EOtC lessons may foster inclusion and tailored learning processes in this context. The analysis is guided by the question of whether the change of location results in different practices and how these differences and their practical consequences can be described in terms of teaching and learning. The didactic triangle [90] serves as a guide when examining the relationships between teacher and pupil, between pupil and subject matter, and between teacher and subject matter.

#### 2.7.2. Lesson characteristics and applied teaching in EOtC.

To assess teaching and learning processes in EOtC the validated, reliable and theory-based observation protocol *Observing Principles of Teaching In School* (OPTIS; Mühlberg et al., under review) will be used to measure the extent of the application of teaching principles during class. With OPTIS, the application of 27 teaching principles, including 39 subordinate observation categories can be observed. As recommended by Mühlberg et al. (under review), a 6-point-rating scale will be used during observations, from “+++” (6 “does apply completely”) to “- - - “(1 “does not apply at all”). Observation intervals are determined by changes in the social form during teaching. For each interval, one observation protocol must be assessed. Whenever the social form shifts, a new observation interval and consequently a new observation protocol begins. Prior to the observations, all observers will be trained to use OPTIS correctly.

Table 1 provides a comprehensive table listing all survey instruments, dates, frequency and target groups. A pilot test of the questionnaires indicated that the minimum appropriate age for participation is 9 years (3^rd^ grade), as younger pupils had difficulties understanding the intended meaning of the questions.

**Table 1:** Overview of research methods.

Instrument	Purpose	Target Group	Timing & Frequency	Data Type	Research Area
EOtC experience	EOtC background	Pupils	Once p. school year	Quantitative	All
SES	Objective Socioeconomic Status	Parents and guardians	Once p. school year	Quantitative	All
SSS	Subjective Socioeconomic Status	Pupils	Once p. school year	Quantitative	All
STSE	Teachers’ self-efficacy	Teachers	Once p. school year	Quantitative	All
Teaching experience	Years of teaching experience	Teachers	Once p. school year	Quantitative	All
2-MEV	Appreciation of nature	Teachers and pupils	1^st^ and 2^nd^ half of the school year	Quantitative	Ecological
Ecological mapping	Biodiversity and structural complexity of outside learning environments	–	Once p. school year	Quantitative	Ecological
Species knowledge	Knowledge of species	Teachers and pupils	1^st^ and 2^nd^ half of the school year	Quantitative	Ecological
IINS & NCI	Nature connectedness	Teachers and pupils	1^st^ and 2^nd^ half of the school year	Quantitative	Ecological and Health
ActiGraph^®^	PA and SB	Teachers	4 days, during cortisol samples	Quantitative	Health
BPN-CS	Fulfillment of BPN	Pupils	Once p. school year	Quantitative	Health
EPOCH-G-S	School-related well-being	Pupils	Once p. school year	Quantitative	Health
Cognitive Interviews	Validation of EPOCH-G-S for younger pupils	Pupils	Once p. school year	Qualitative	Health
COPSOQ III	WRQoL	Teachers	Once p. school year	Quantitative	Health
EEG	Cognitive performance	Pupils	Once p. school year: 1 EOtC day and 1 regular day	Quantitative	Health
PANAS	Emotional well-being	Teachers	2^nd^ half of the school year: 5x p. day, 2 EOtC days and 2 regular days	Quantitative	Health
rMEQ	Sleep chronotype	Teachers	Once p. school year	Quantitative	Health
Salivary cortisol	Physiological stress	Teachers	2^nd^ half of the school year: 5x p. day, 2 EOtC days and 2 regular days	Quantitative	Health
Stress scale	Subjective stress level	Pupils	Once during EEG measurement	Quantitative	Health
SQS	Sleep quality	Teachers	Each morning with cortisol samples	Quantitative	Health
OPTIS	Instructional quality/ applied teaching principles	Teachers and pupils	Once p. school year: 1 EOtC day and 1 regular day	Quantitative	Pedagogy
Video and audio recordings	Teaching and learning analysis	Teachers and pupils	Once p. school week: 1 EOtC day and 1 regular day	Qualitative	Sociology & School Pedagogy

### 2.8. Data analysis

#### 2.8.1. Statistical analysis.

To evaluate the quantitative data of WRQoL, emotional well-being, psychological well-being, nature connectedness, appreciation of nature, PA and SB descriptive analyses will be carried out, in order to present central tendencies and distributions of the variables collected. Inferential statistical methods and repeated measures analysis of variance will be used to test group differences and hypotheses. To make sure the data structure enables this procedure, the data will be checked for normal distribution and homogeneity of variance before the analysis.

To investigate the relationship between the ecological features of the learning environments and other study variables correlation analyses, linear regression models and possibly generalized linear models or mixed-effect models will be used in order to take possible hierarchical structures of the data into account.

Linear mixed models will be used for the cortisol measurements to adequately account for intra-individual changes and covariates such as sleep quality or chronotype. In addition to descriptive analyses of the collected data, Bayesian Hierarchical Linear Models will be used to investigate the influence of PA and SB on cortisol levels.

#### 2.8.2. Analysis of qualitative data.

Interviews and focus groups will be recorded, followed by transcription and coding using Max Weber Qualitative Data Analysis (MAXQDA) software. Coding and the development of a category system will be performed by two independent researchers and consultation between them will take place frequently to discuss discrepancies.

MAXQDA will also be used to analyse the film and audio recordings, and the transcripts created will form the database for all further analysis. Sequence analysis of the transcripts is used to examine both the teaching practices and interactions of the teachers as well as the behaviour and communication of the pupils. Through the reconstructive analysis of the influences of the natural environment, the situations it evokes and how the actors within the teaching process react upon them are investigated. In a second step the effects can be assessed regarding the parameters of teaching.

#### 2.8.3. Research ethics.

All planned measurements and data analyses were reported to the ethics committees of the Technical University of Munich (reference number 2025–73-NM-BA) and the Julius-Maximilians-Universität Würzburg (reference number EK_JMU_HF_PSPS_2026_12) and verified for ethical compliance.

## 3. Discussion

The aim of this methods paper was to present a large-scale, interdisciplinary research project on EOtC in Germany. By describing the disciplines involved, as well as the survey methods, the analysis strategies, and the planned timeline, the paper aims to provide a comprehensive overview of the study concept. The interdisciplinary nature of the project enables us to examine the multifaceted effects of EOtC on health, social interaction, pedagogical processes, and ecological aspects of all participants within a comprehensive context. While the exploratory, interdisciplinary approach offers several advantages, the study specifically follows a parallel multi-strand research approach, combining independent quantitative and qualitative sub-studies conducted within the same overarching project. The quantitative component is based on a multi-level, predominantly cross-sectional design and was chosen to reflect authentic school conditions with minimal disruption to everyday teaching practice, thereby ensuring high ecological validity. At the same time, it enables comparability through the use of control groups where feasible and intra-individual approaches where experimental control is limited. The qualitative component follows a Grounded Theory approach based on video data, aiming to reconstruct processes of teaching and learning in outdoor contexts. Both strands address distinct research questions and are primarily analysed independently. This approach offers several advantages but also comes with certain limitations, which are discussed later in the discussion.

The chosen design represents a central strength of the project. It enables the inclusion of all disciplines involved and the survey measures, such as the observation of teaching characteristics (OPTIS) and the video-based analysis of social interactions between teachers, pupils, the EOtC-learning environment, and teaching objects. To our knowledge, this is the first EOtC study to document and examine EOtC teaching in such detail. In addition, the selected ecological investigations have not yet been carried out in this depth, with the intensive examination of the EOtC-learning environments representing a particular novelty in the field. This allows observed effects to be linked retrospectively to specific teaching processes.

Another strength of the project lies in the consistent involvement of teachers. Previous EOtC research has focused primarily on the perspective and effects on pupils, while the role and experience of teachers have received little attention. Many of the effects observed in the classroom are closely linked to teachers’ own well-being, which is often overlooked. By focusing on teachers in this study, the potential influences of EOtC on teachers’ health, connection to nature, lesson structure and workload can be better understood.

The value of this approach lies in relating different perspectives to one another, thereby highlighting not only isolated findings but also their broader practical relevance. Social science studies that investigate the effects of EOtC on students and teachers may not investigate the actual ecology and biodiversity within the learning site. On the other hand, studies that investigate the ecology of such spaces in urban and rural areas, may not investigate the physical and mental health effects of such spaces on pupils and teachers. Such an approach as this one specifically relates the outdoor environment’s characteristics with the health, wellbeing and knowledge of people learning and teaching in these spaces.

At the same time, this paper also reflects the diverse requirements that an educational concept must meet in order to be sustainable within the complex German school system. Studies on the sustainability of school interventions to promote student health highlight that add-on programs which are not integrated into day-to-day teaching often only have limited effects and cannot be established in the long term, as they have little curricular integration and often require significant time and human resources [91]. In contrast, EOtC is designed to be seamlessly embedded into the regular curriculum, making it a feasible and sustainable approach within existing school structures. By addressing these structural requirements, EOtC has the potential to offer real added value to both teachers and pupils. This comprehensive analysis strengthens the significance of the results and lays the foundation for a better understanding of the implementation of EOtC in real teaching situations.

Nevertheless, certain limitations need to be acknowledged. While randomized controlled trials are considered the gold standard when it comes to proving the causal effects of interventions [92], such a study design was neither practical nor desirable in the context of EOtC. The aim of the project is to investigate the effects of EOtC under real-world conditions, while minimising interference with regular teaching routines. Randomly assigning classes to intervention or control groups would have had a profound impact on school organization and influenced possible effects. Instead, a study design was chosen that allows for both intra- and inter-individual comparisons without disrupting or distorting the normal daily routine of schools and their implementation of EOtC.

Despite the possibilities for intra- and interindividual comparisons, differences at the class or school level cannot be fully controlled. In addition, there are external influencing factors such as family background or parental support, which also shape school learning, appreciation and connection to nature, and, of course, children’s health, and can thus lead to variances in the results. To account for such influences, we plan to collect data on selected background variables, such as the children’s previous EOtC experience in kindergarten or school, their SSS, or the teachers’ sleeping rhythms and teaching experience, which allows us to at least estimate and consider potential confounding factors. While such factors pose a challenge to comparability, they also reflect the real complexity of school environments in which EOtC is embedded.

In the light of current social and educational challenges, schools are increasingly faced with the challenge of designing learning processes in a way that considers not only cognitive and academic goals but also health, social, and environmental development aspects. Given that schools constitute one of the main life contexts in which children and teachers spend a large part of their time, they inevitably shape not only learning but also health, social relations, and opportunities for experiencing nature. EOtC offers a promising approach to addressing these issues simultaneously. While EOtC is already firmly established in the curricula of several countries, its implementation in Germany is still in its early stages and depends on the commitment of individual teachers. Through its interdisciplinary perspective and consistent consideration of both pupils and teachers, this project not only strengthens the international evidence base on EOtC but also provides urgently needed insights into its applicability within the German school system.

## 4. Conclusion

In conclusion, this study presents a large-scale, interdisciplinary project that comprehensively examines the effects of EOtC in Germany. In contrast to previous studies, there is a particular focus on the role of teachers and on the detailed analysis of teaching processes in connection with ecological conditions. With a mixed methods design that allows for intra- and interindividual comparisons and works under conditions that are as realistic as possible, the project opens new perspectives on the connections between health-related and ecological aspects, as well as aspects of social interaction and pedagogy. In doing so, it contributes to international EOtC research, closes key research gaps in the German school system, and creates a base for sustainable approaches that can support both teachers and pupils, thereby also fostering principles of Education for Sustainable Development.
