# Supplementary material for: Education outside the classroom in Germany: An interdisciplinary multi-methods Study Protocol
Source: PLoS One. 2026 Jun 23;21(6):e0337524. doi: 10.1371/journal.pone.0337524 (PMC13289885; doi:10.1371/journal.pone.0337524)
Supplement: Attachment — Submitted filename: Response to Reviewers.docx [file pone.0337524.s003.docx]

POINT-BY-POINT RESPONSE

*Submission ID PONE-D-25-57440*

We thank the editor and reviewers for reading and reviewing our manuscript and appreciate their helpful comments. We hope that we have addressed the concerns in an appropriate manner and thus improved our manuscript. All comments raised in the manuscript are listed below, together with their corresponding locations in the text and our individual responses.

EDITOR:

**The author mentions that resonance theory will be utilized to investigate if EOtC promotes moments of resonance and diminishes feelings of alienation. However, there is no explanation given regarding the specifics of resonance theory, including its principles and tenets. It is important for the author to provide a detailed description of resonance theory and clarify its relevance to the study.**

Thank you for this important comment. We have revised the manuscript to include a detailed explanation of resonance theory and its core principles. Furthermore, we clarify how these dimensions are applied in the context of this study by using them as sensitizing concepts to analyse empirical video data. This addition also makes the relevance of resonance theory for investigating interaction patterns and teaching–learning processes in EOtC more explicit (page 8-9, lines 203 – 214).

**The research questions need to be more clearly articulated. Additionally, the method section requires further development. Specific corrections can be found in the attached reviewed manuscript.**

Thank you for this valuable feedback. We have revised the manuscript accordingly. The research questions have been fully specified, structured, and clearly articulated in a dedicated section at the end of the Introduction. In addition, the Methods section has been thoroughly revised in line with all specific comments provided in the annotated manuscript. All suggested clarifications and improvements have been addressed in detail.

REVIEWER 1:

**This is a very interesting multidisciplinary study exploring education Outside the Classroom in Germany. It is important for the author to provide full names first and abbreviate afterwards. For an example WRQoL , EEG and MAXQDA have to be written in full first and abbreviated going forward to enable your readers to follow and understand.**

Thank you very much for your feedback. We have carefully checked all used abbreviations and made sure the full names are provided first, before the abbreviations are used.

**Keywords are missing in the abstract. Keywords are critical for optimizing searchability in academic databases, increasing the visibility of research, improving discoverability and facilitating proper indexing by search engines.**

Thank you for this valuable feedback. We have made sure that all key words are included in the abstract, in the exact wording.

**The author states that resonance theory will be used to explore whether EOtC fosters
moments of resonance and reduces experiences of alienation. However, no explanation is provided on what resonance theory entails, its tenets, principles etc.**

Thank you for highlighting this point. We have expanded the manuscript to provide a clearer and more comprehensive description of resonance theory, including its central dimensions and underlying principles. In addition, we explicitly outline how the theoretical framework informs our analytical approach by guiding the examination of interaction patterns and learning processes in the empirical data. This revision ensures that the theoretical foundation and its relevance to the study are clearly articulated (page 8-9, lines 203 – 214).

REVIEWER 2:

**Good article and study, however the research questions are not clearly written. There is a need to work on the method section. Find specific correction in the attached reviewed manuscript.**

Thank you for your positive evaluation of the manuscript and for your helpful comments. We have substantially revised the manuscript to address your concerns. The research questions have been clearly reformulated, structured by thematic domains, and explicitly included in the Introduction. Furthermore, the Methods section has been revised comprehensively in response to the detailed comments provided in the annotated manuscript, ensuring improved clarity and methodological transparency.

**EOtC (page 12, line 96)**

Thank you for this feedback, we have replaced this.

**What levels? (page 13, line 105)**

Thank you very much for this valid point. We have revised the misleading wording at the end of the sentence and ensured that it is now clear that we are referring to the interrelationship between the disciplines mentioned.

**Going through the instruments, this study is employing mixed method of research approach. Reflect that in this section and mention the type of mixed method. (page 13, lines 121-122)**

Thank you for this helpful suggestion. We have revised the study design section to explicitly describe the research approach used in this study and clarified the overall methodological framework in greater detail. More specifically, we now describe the study as following a parallel multi-strand research approach, combining independent quantitative and qualitative sub-studies within an overarching interdisciplinary framework. We hope that these revisions provide a clearer understanding of the methodological structure of the project and allow the interdisciplinary character of the study to emerge more strongly. (page 7, lines 156 – 158).

**Is this the inclusion criteria? Be specific about the inclusion and exclusion criteria. (page 16, lines 185-188)**

Thank you for this helpful comment. We have clarified that the described threshold constitutes an inclusion criterion by explicitly stating that schools were included if they implemented EOtC for at least four hours every two weeks. We have also specified that no additional exclusion criteria were defined (page 9-10, lines 231-236).

**This was not stated clearly earlier. (page 16, line 200)**

Thank you very much for your very valid comment. We have ensured that the text in the upper section clearly states that these are the inclusion criteria, so hopefully there will be no further uncertainty at this moment (page 9-10, lines 231-236).

**whats the approval number for this (page 30, line 497)**

Thank you for pointing out the missing number for the second ethics application. We have corrected the relevant section (page 24, line 544).

**mixed method is more of research approach than design. Also which type is being used? You can then mention the design used for qualitative phase and also the one for quantitative phase. Also explain why each design was chosen for each phase (page 30, line 506)**

Thank you for this helpful comment. We agree that “mixed methods” is more appropriately described as a research approach rather than a specific design. We have therefore revised the manuscript accordingly and clarified the overall approach.

More specifically, we now describe the study as following a parallel multi-strand research approach, in which independent quantitative and qualitative sub-studies are conducted concurrently within an overarching interdisciplinary framework. In addition, we have specified the designs of the individual quantitative and qualitative components and also briefly clarified the rationale for these choices (pages 24-25, lines 552-563).

JOURNAL REQUIREMENTS:

**1. Please ensure that your manuscript meets PLOS ONE's style requirements, including those for file naming.**

Thank you for this comment. We have carefully reviewed the manuscript and all associated files to ensure compliance with PLOS ONE’s style requirements, including file naming conventions. The figure files have been corrected and appropriately labeled.

**2. Your ethics statement should only appear in the Methods section of your manuscript. If your ethics statement is written in any section besides the Methods, please delete it from any other section.**

Thank you for this reminder. We have deleted the ethics statement from the declarations.

**3. Please provide a complete Data Availability Statement in the submission form, ensuring you include all necessary access information or a reason for why you are unable to make your data freely accessible. If your research concerns only data provided within your submission, please write "All data are in the manuscript and/or supporting information files" as your Data Availability Statement.**

Thank you for this comment. A complete Data Availability Statement has now been provided in the submission form. In addition, the statement has been included at the end of the manuscript. The statement clarifies that quantitative data will be made publicly available upon study completion and after full anonymization, while qualitative data, such as videos or interviews cannot be shared publicly due to ethical and legal constraints related to potential identifiability. It further specifies the conditions under which fully anonymized data may be accessed upon reasonable request and with approval from the ethics committee responsible.

**4. Please upload a copy of Figure 1, 2, to which you refer in your text on page 8. 9. If the figure is no longer to be included as part of the submission please remove all reference to it within the text.**

Thank you for pointing this out. Due to an error during the submission process, the figures were incorrectly labeled in the system (Figures 1 and 2 were interchanged). This has now been corrected, and Figure 1 has been properly uploaded and labeled accordingly. All figure references in the manuscript have been checked and updated where necessary to ensure consistency.

**5. We note that Figure 1 in your submission contain [map/satellite] images which may be copyrighted. We require you to either (1) present written permission from the copyright holder to publish these figures specifically under the CC BY 4.0 license, or (2) remove the figures from your submission.**

Thank you for highlighting this important issue. The original figure included map material based on proprietary data (Google Maps. We have therefore removed the original image and replaced it with a newly created figure based on publicly available, non-copyrighted data by Natural Earth. The revised figure complies fully with the journal’s licensing requirements and has been adapted for the purposes of this study. The figure caption has been updated accordingly to reflect the data source and licensing.

**6. If the reviewer comments include a recommendation to cite specific previously published works, please review and evaluate these publications to determine whether they are relevant and should be cited. There is no requirement to cite these works unless the editor has indicated otherwise.**

Thank you for this input. We have checked the citations again and made sure to include specific works.

**7. Please review your reference list to ensure that it is complete and correct. If you have cited papers that have been retracted, please include the rationale for doing so in the manuscript text, or remove these references and replace them with relevant current references.**

Thank you for this reminder, we have checked all references and made sure their citation is correct, they are needed in the manuscript and are not retracted.
